# A novel thymidylate synthase from the *Vibrionales*, *Alteromonadales*, *Aeromonadales*, and *Pasteurellales* (VAAP) clade with altered nucleotide and folate binding sites

**DOI:** 10.7717/peerj.5023

**Published:** 2018-06-15

**Authors:** Alonso A. Lopez-Zavala, Eduardo Guevara-Hernandez, Luz H. Vazquez-Lujan, Arturo Sanchez-Paz, Karina D. Garcia-Orozco, Carmen A. Contreras-Vergara, Gamaliel Lopez-Leal, Aldo A. Arvizu-Flores, Adrian Ochoa-Leyva, Rogerio R. Sotelo-Mundo

**Affiliations:** 1 Biomolecular Structure Laboratory, Centro de Investigación en Alimentación y Desarrollo, A.C., Hermosillo, Sonora, Mexico; 2 Departamento de Ciencias Quimico Biologicas, Universidad de Sonora, Hermosillo, Sonora, Mexico; 3 Laboratorio de Referencia, Análisis y Diagnóstico en Sanidad Acuícola, Centro de Investigaciones Biologicas del Noroeste, Hermosillo, Sonora, Mexico; 4 Laboratorio de Genetica de Plantas, Centro de Investigación en Alimentación y Desarrollo, A.C., Hermosillo, Sonora, Mexico; 5 Departamento de Microbiología Molecular, Instituto de Biotecnología, Universidad Nacional Autónoma de México, Cuernavaca, Morelos, Mexico

**Keywords:** *Vibrio parahaemolyticus*, Trimethoprim, Gammaproteobacteria, Alteromonadales, Enzyme, Aeromonadales, *Pasteurellales*, Thymidylate synthase

## Abstract

Thymidylate synthase (TS, E.C. 2.1.1.45) is a crucial enzyme for *de novo* deoxythymidine monophosphate (dTMP) biosynthesis. The gene for this enzyme is *thyA*, which encodes the folate-dependent TS that converts deoxyuridine monophosphate group (dUMP) into (dTMP) using the cofactor 5,10-methylenetetrahydrofolate (mTHF) as a carbon donor. We identified the *thyA* gene in the genome of the *Vibrio parahaemolyticus* strain FIM-S1708+ that is innocuous to humans but pathogenic to crustaceans. Surprisingly, we found changes in the residues that bind the substrate dUMP and mTHF, previously postulated as invariant among all TSs known (Finer-Moore, Santi & Stroud, 2003). Interestingly, those amino acid changes were also found in a clade of microorganisms that contains *Vibrionales*, *Alteromonadales*, *Aeromonadales*, and *Pasteurellales* (VAAP) from the *Gammaproteobacteria* class. In this work, we studied the biochemical properties of recombinant TS from *V. parahemolyticus* FIM-S1708+ (VpTS) to address the natural changes in the TS amino acid sequence of the VAAP clade. Interestingly, the *K_m_* for dUMP was 27.3 ± 4.3 µM, about one-fold larger compared to other TSs. The *K_m_* for mTHF was 96.3 ± 18 µM, about three- to five-fold larger compared to other species, suggesting also loss of affinity. Thus, the catalytic efficiency was between one or two orders of magnitude smaller for both substrates. We used trimethoprim, a common antibiotic that targets both TS and DHFR for inhibition studies. The IC_50_ values obtained were high compared to other results in the literature. Nonetheless, this molecule could be a lead for the design antibiotics towards pathogens from the VAAP clade. Overall, the experimental results also suggest that in the VAAP clade the nucleotide salvage pathway is important and should be investigated, since the *de novo* dTMP synthesis appears to be compromised by a less efficient thymidylate synthase.

## Introduction

Pathogens comprise a highly complex and diverse community of organisms that include viruses, bacteria, fungi, protozoans, parasitic arthropods, mollusks, worms, and few chordates (Bergh et al., 1989; Suttle, 2005; Kristensen et al., 2010). Because of the constant adverse conditions imposed by their hosts to escape infection, pathogens rapidly evolve sophisticated strategies to evade or subvert the host immune response after its establishment (Aguileta et al., 2009).

Bacterial infections from Vibrio are still a critical problem in human health (Colwell, 1996), and also to aquatic organisms (Holmström et al., 2003; Wang, Li & Lin, 2008).

Bacteria from the genus *Vibrio* are part of the natural microflora of wild and cultured shrimp species (Gomez-Gil et al., 1998; Esiobu & Yamazaki, 2003; Liu et al., 2011; Cornejo-Granados et al., 2017), although some species, as *V. harveyi*, *V. alginolyticus*, *V. anguillarum*, *V. vulnificus*, *V. splendidus* and *V. parahaemolyticus* have usually been associated with diseases in shrimp and shellfish (Lavilla-Pitogo, 1995; Sung et al., 2001; Hsu & Chen, 2007; Kumar et al., 2014; Cornejo-Granados et al., 2017).

By the end of 2009, the emergence of a new bacterial pathogen affected shrimp aquaculture in China and later spread to Vietnam, Malaysia, and Thailand (Leaño & Mohan, 2012; De Schryver, Defoirdt & Sorgeloos, 2014). Eventually, the disease reached the Western Hemisphere and emerged in Mexico in early 2013 (Nunan et al., 2014). Several studies have demonstrated that the etiological agent of this disease, initially referred as Early Mortality Syndrome or EMS, and more recently named Acute Hepatopancreatic Necrosis Disease or AHPND (Lightner et al., 2012) were specific strains of *V. parahaemolyticus* that do not contain neither the human pathogenic markers *tdh* (thermostable direct hemolysin) nor *trh* (tdh-related hemolysin) (Tran et al., 2013; Joshi et al., 2014; Soto-Rodriguez et al., 2015). In fact, the AHPND-causing strains of *V. parahaemolyticus* harbor a 70 kbp plasmid, which encodes two toxins, *ToxA* and *ToxB*, homologs of the *Photorhabdus* insect-related (Pir) toxins *PirA* and *PirB* (Lee et al., 2015). Currently, there are no active treatments available against this toxigenic Vibrio strains, and there is a need for of antibacterial strategies. As with any other living organism, during the bacterial division cycle, the pools of nucleotides must be maintained at relatively constant concentrations, and nucleotide synthesis is greatly stimulated (Lane & Fan, 2015). Therefore, this pathway could be targeted with antibacterial compounds.

Thymidylate synthase (EC 2.1.1.45, TS) catalyzes the reductive methylation of 2′-deoxyuridine-5′-monophosphate (dUMP) to 2′-deoxythymidine-5′-monophosphate (dTMP) and dihydrofolate, assisted by the co-factor 5,10-methylenetetrahydrofolate (mTHF) (Carreras & Santi, 1995). This reaction is the final step in the only *de novo* synthetic pathway to thymidylate, so it is essential for DNA replication. *thyA* corresponds to the folate-dependent TS found in *E. coli* (Belfort et al., 1983), invertebrates, vertebrates and humans. Recently, a novel flavin-dependent TS was described as *thyX*. (Myllykallio et al., 2002), which also has been postulated as an antibacterial target (Choi, Karunaratne & Kohen, 2016).

Folate-dependent TS is an excellent drug target for cancer cells, and there are examples of drugs in clinical use (Jarmula, 2010). TS is also a good target for antibacterial infectious diseases (Ferrari, Costi & Wade, 2003). The folate-dependent TS (*thyA*) has been thoroughly studied in bacteria to a structural detail where the function of every active-site amino acid residue has been determined (Finer-Moore, Santi & Stroud, 2003). Nonetheless, with the advent of massive genome sequencing, changes in bacterial *thyA*-conserved residues have been identified. For example, changes in charged residues close to the active site have been found in *Wigglesworthia glossinidia* TS (Garg et al., 2015). The importance of electrostatics in the TS active site was demonstrated by successive mutagenesis of *E. coli* TS towards the charged residues in *W. glossinidia*, observing important changes in the kinetic parameters of the mutants (Garg et al., 2015). Importantly, *V. parahaemolyticus* strain FIM-S11708+ TS (Gomez-Jimenez et al., 2014) has a change to glycine in one of the four arginines that coordinate the nucleotide-phosphate group.

Indeed, little is known regarding the biochemical properties, functionality and metabolic role of TS on Gammaproteobacteria or Vibrionales whatsoever that are pathogenic to humans or marine organisms. Therefore, this study aimed to investigate the biochemical properties of the TS from a *V. parahaemolyticus* toxigenic strain (VpTS). Comparison of the Michaelis–Menten kinetic parameters, enzyme inhibition with a known antibacterial such as trimethoprim and molecular modeling provides insight into the importance of the *de novo* dTMP biosynthesis into the VAAP clade.

## Materials and Methods

### Genome used and thymidylate synthase orthologous genes

We used the thymidylate synthase *Vibrio parahaemolyticus* (GenBank WP_100088861.1) found in the FIM-S1708+ strain genome deposited as GenBank JPLV00000000.1 (Gomez-Jimenez et al., 2014) as a seed to identify the homologous TS, using blast searches with an *E*-value cutoff of 1.0^e−15^ against the proteomes encoded in 38 genomes belonging to the gamma-proteobacteria class (File S1). All proteins that shown an alignment ≤70% of their length were kept for further analysis. We collected these genomes for two reasons: First, to get a better perspective of the variation of the TS across the Gammaproteobacteria (primarily focused on the active site) and second, these genomes have been used in phylogenomic analyses of that bacterial class (Williams et al., 2010).

### Phylogeny among the thymidylate synthase

To conduct a Maximum Likelihood (ML) phylogeny, we created a protein Multiple Sequence Alignment (MSA) using MUSCLE with 50 iterations (Edgar, 2004). We ran the ML phylogeny specifying the LG+G+I model, as determined by ProtTest3 (Darriba et al., 2011) and using non-parametric bootstrap analysis (100 replicates) to establish the support for the clades. The phylogenetic tree was colored and edited using FigTree v1.4.3 (http://tree.bio.ed.ac.uk/software/figtree/).

### VpTS expression and purification

VpTS was expressed in *Escherichia coli* using a codon-optimized synthetic gene cloned into the T7-promoter expression vector pJexpress414 which contains the ampicillin resistance gene (DNA2.0). We used the amino acid sequence of *Vibrio parahaemolyticus* thymidylate synthase (GenBank WP_100088861.1) including in the N-terminus a 10 His-tag and the cutting site for the PreScission Protease (GE Healthcare, Little Chalfont, UK). The total theoretical mass would be 35,944 Da and the tag could be removed upon treatment with the protease.

The *E. coli* BL21(DE3)-SI strain was used to express VpTS. To facilitate yield of soluble protein we used a plasmid containing the chaperones groES-groEL-Tig (TAKARA plasmid PG-Tf2). For this, the bacteria were transformed first with plasmid pPG-Tf2 with chloramphenicol selection at 20 µg/mL, and later with the pJexpress414-VpTS plasmid using chloramphenicol and ampicillin at 100 µg/mL. Both antibiotics were used in all further procedures.

A 5 mL of a starter culture of transformed bacteria was incubated overnight and used to inoculate 1 L of LB broth without NaCl. The bacteria were incubated in an orbital shaker at 250 rpm at 37 °C. When an optical density of 0.4 at 600 nm was obtained, chaperone expression was induced by addition of tetracycline to a final concentration of 9 ng/mL. When optical density reached 0.6 units (*λ* = 600 nm), the T7-promoter was induced with IPTG and NaCl to a final concentration of 0.1 mM and 0.3 M respectively. Cell growth was continued for 24 h at 25 °C. The bacterial pellet was obtained by centrifugation at 7,500× g at 4 °C, and the biomass was lysed by sonication in 20 mM potassium phosphate buffer pH 7.5, 5 mM dithiothreitol (DTT), 0.5 mM phenyl methyl sulfonyl fluoride (PMSF), and 5 mM benzamidine. The bacterial lysate was clarified at 25,000× g for 20 min at 4 °C.

For metal affinity chromatography, a 5 mL Ni-His Trap column (GE Healthcare) was equilibrated with 20 mM phosphate buffer pH 7.5, 0.5 M NaCl (buffer A) in an Äkta Prime chromatographer (GE Healthcare) at 1 mL/min. The clarified lysate was loaded into the column and washed with at least five volumes of buffer A until the absorbance at 280 nm returned to baseline. Elution was done with a linear gradient of buffer A and buffer A plus 500 mM imidazole. Fractions of 3 mL were collected. Purification was followed by 12% SDS-PAGE using pre-casted TGX stain-free gels (BioRad) and images were recorded on a GelDoc Easy Imaging system (BioRad). The TGX stain-free detection system has a sensitivity comparable to silver staining (Gilda & Gomes, 2013).

To further purify VpTS and confirm the oligomeric state, size exclusion chromatography was used using a Superdex 75 10/300 column in an Äkta Pure chromatographer (GE Healthcare) at 1 mL/min with Tris HCl 20 mM pH 7.5 and NaCl 100 mM as running buffer. Molecular weight (MW) standards were aprotinin (6.5 kDa), ribonuclease A (13.7 kDa), carbonic anhydrase (29 kDa), ovalbumin (44 kDa), conalbumin (75 kDa) and Blue Dextran (2,000 kDa) to determine the column void volume. VpTS native molecular weight was calculated from linear regression of a *K*_*av*_ *vs*. log MW plot, using the formula

\begin{eqnarray*}{K}_{av}=({V}_{t}-{V}_{o})/({V}_{f}-{V}_{o}) \end{eqnarray*}

where *V*_*t*_ is the total column volume, *V*_*o*_ is the void column and *V*_*f*_ is the volume where the sample or a molecular weight standard is eluted. Fractions were analyzed by 12% SDS-PAGE as mentioned above. Purified protein was quantitated using the bicinchoninic acid method (BCA kit; Pierce, Waltham, MA, USA).

### VpTS kinetics and IC_50_ inhibition

TS kinetics were determined using a spectrophotometric assay following the formation of dihydrofolate at 340 nm (ε_340nm_ = 6,400 M^−1^ cm^−1^) in a CARY-50 (Varian) UV-vis spectrophotometer as previously described (Arvizu-Flores et al., 2009). The reaction buffer contained 50 mM Tris-HCl pH 7.5, 5 mM DTT, 1 mM EDTA, 25 mM MgCl_2_. The substrates were mTHF (Schircks Laboratories, Jona, Switzerland) and dUMP (Sigma-Aldrich, St. Louis, MO, USA). The assay was done in a final volume of 1 mL at 30 °C. The reaction was started by addition of 0.3 µM of recombinant VpTS, and the initial rates were determined by recording the absorbance increase at 340 nm, changing the substrate concentration for mTHF (0–300 µM) and dUMP (0–150 µM). Experimental data were obtained in triplicate and fitted to the Michaelis–Menten equation by a non-linear regression analysis using the GraFit software (Erithacus Software).

Steady-state IC_50_ inhibition was determined with trimethoprim (TRIM, Sigma-Aldrich, St. Louis, MO, USA) as an antifolate in standard activity conditions (Arvizu-Flores et al., 2009) with saturating substrate concentrations (mTHF 300 µM and dUMP 150 µM) by triplicate. Data were adjusted to a dose–response model using non-linear fit with the GraFit software.

### Structure modeling

The VpTS structural model was built using Protein Homology/analogY Recognition Engine V Phyre2.0 server (Kelley et al., 2015). The best model was selected by the highest sequence identity, alignment coverage and model confidence factor within the closed conformation structures. The most similar bacterial structure in the dUMP-bound closed conformation, was *E. coli* TS (32% of sequence identity; PDB 1AXW) as the reference structure. The structural analysis was performed in CCP4MG V7.0 program (McNicholas et al., 2011)

## Results

### Expression and purification of VpTS

**Figure 1:**
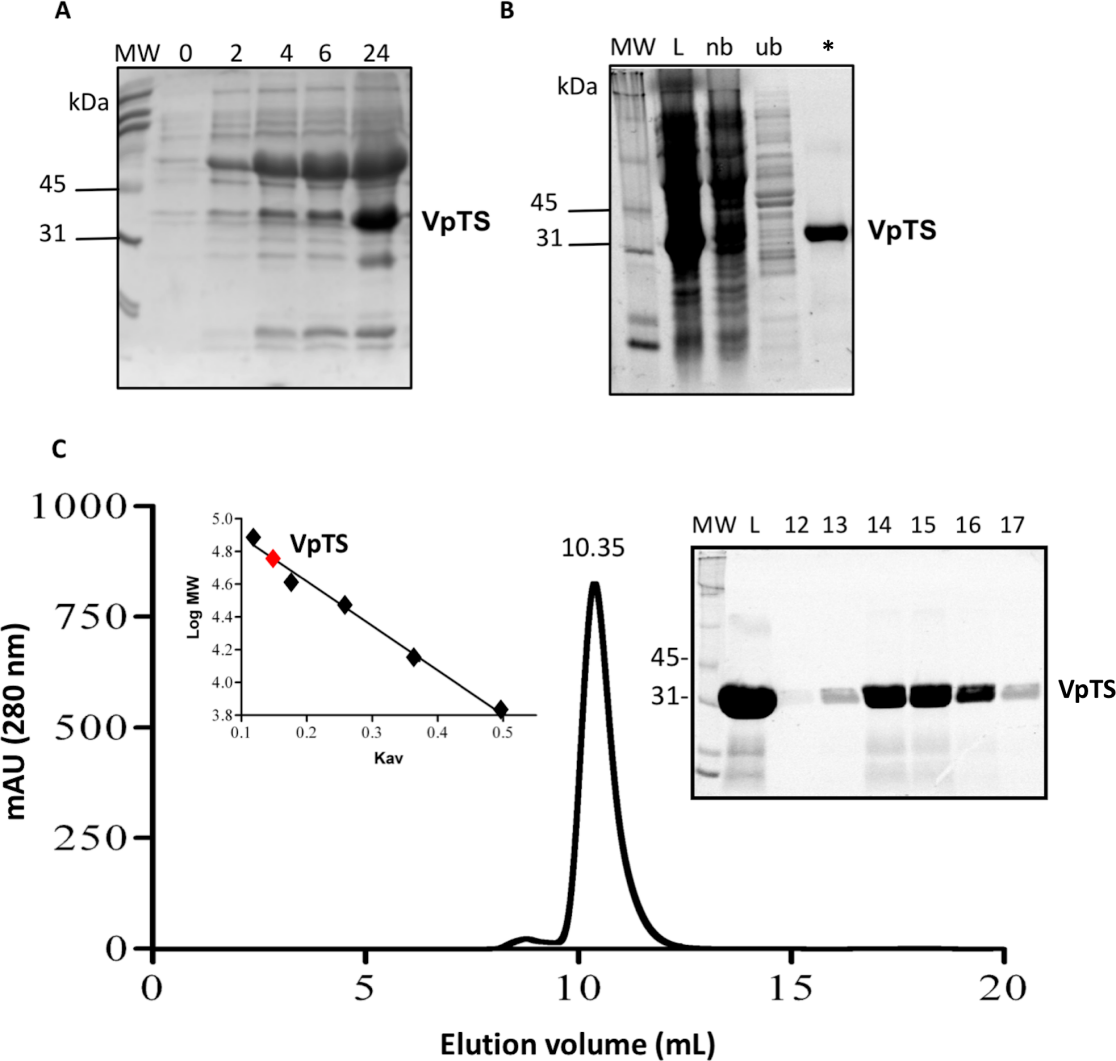
Recombinant expression and purification of VpTS. SDS-PAGE was done in BioRad TGX 12% Stain Free precast gels. 20 mL of protein sample were treated with the same volume of 2X SDS-Sample buffer, heated 5 min a 95 °C and loaded per lane. (A) SDS-PAGE of bacterial lysates from expression in LB media. Lanes represent 0, 2, 4, 5 and 24 h after induction with IPTG. (B) Protein purification follow-up using nickel His-tag affinity chromatography. Lane L represents a sample of the bacterial lysate loaded into the column. Lane nb comes from non-bound proteins. Lane ub corresponds to unspecific bound proteins eluted with 10 mM imidazole and the asterisk (*) lane corresponds to VpTS eluted with 375 mM imidazole. (C) Size Exclusion Chromatography. The chromatogram recorded at 280 nm is included showing the volume where VpTS eluted (10.35 mL). As inset, the SDS-PAGE of selected fractions corresponding to the peak are included. M stands for the molecular weight markers, L for the sample loaded and 12–17 corresponding to the fractions around the peak at 10.35 mL. Second inset, the *K*_*av*_ *vs*. log MW plot is shown, where the VpTS native molecular weight mass was interpolated to 70 kDa.

VpTS was co-expressed with the set of chaperones groES-groEL-Tig in *E. coli* strain BL21-SI, observing that the protein was best expressed at 24 h post induction. This was observed in the SDS-PAGE follow-up of expression, where VpTS migrated to an approximate mass of 35 kDa (Fig. 1A). This value is within the range of the theoretical molecular weight of 35,944 Da taking into account the 10-His tag. Besides VpTS, there was a highly co-expressed protein at 60 kDa corresponding to the GroEL chaperone molecular weight (Fig. 1A).

VpTS was purified by IMAC chromatography purification as seen in the SDS-PAGE of the chromatography fractions (Fig. 1B). VpTS eluted at an imidazole concentration of 375 mM, as observed VpTS in the asterisk lane of Fig. 1B. The yield of VpTS after IMAC chromatography was 40 mg of protein per liter.

We also included an extra purification step using size-exclusion chromatography, to confirm the native dimeric TS quaternary structure. A molecular weight of 70 kDa was interpolated from the analysis of the after mentioned calculation (Fig. 1C). The gel filtration purified protein had the same specific TS activity, therefore for enzymatic assays we used VpTS purified by IMAC chromatography only. This has been found also for varicella zoster TS, confirming that IMAC was sufficient for VpTS kinetic studies (Hew et al., 2015). Instead of Coomassie Blue or silver staining, TGX stain-free 12% polyacrylamide gels (BioRad) were used during all the purification steps, since they provide a good method to validate protein purity (Gilda & Gomes, 2013; Rivero-Gutiérrez et al., 2014).

### Enzymatic and inhibition assay

**Figure 2:**
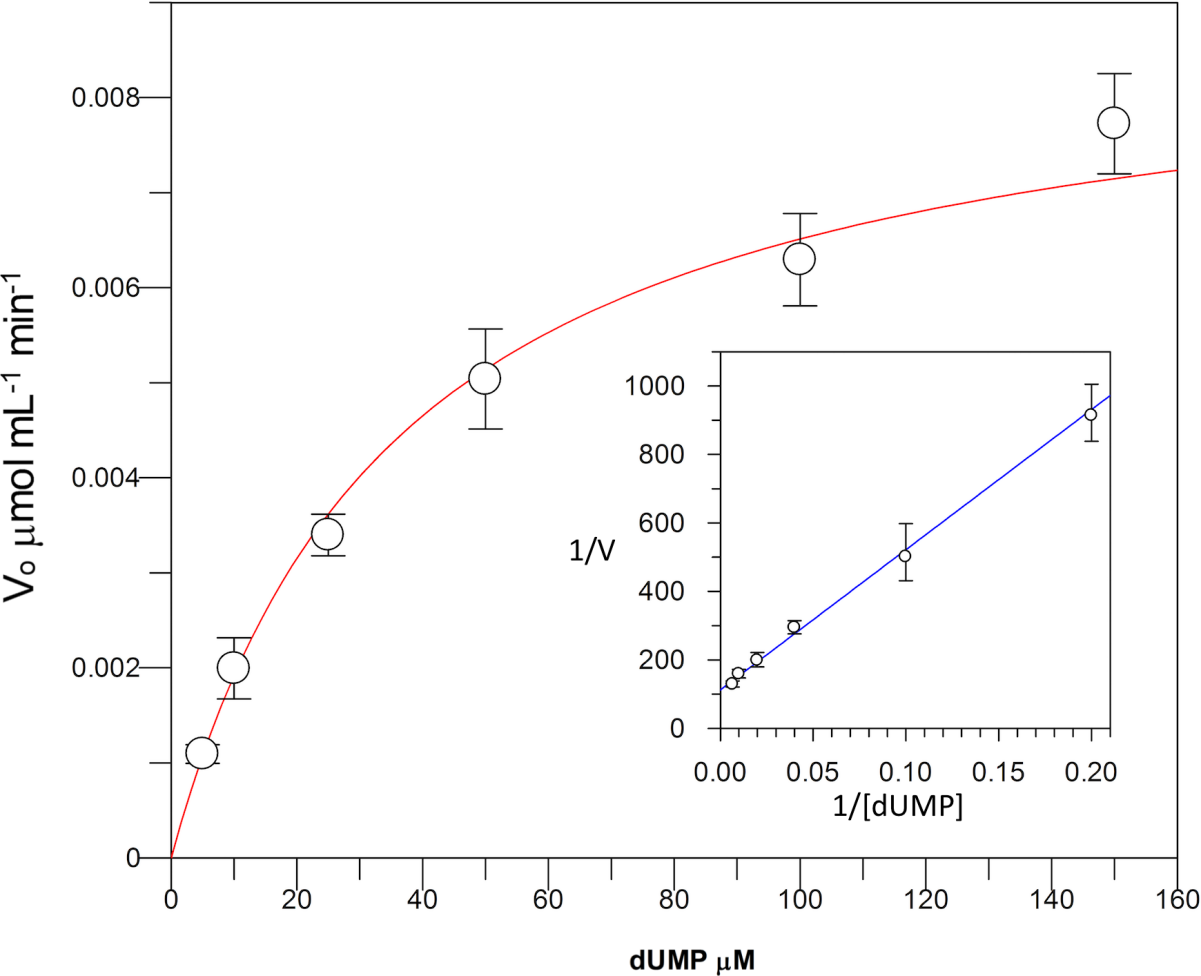
Michaelis-Menten kinetics for VpTS using dUMP as substrate. Initial velocities were measured by triplicate and kinetic parameters were obtained by non-linear fitting with robust weighting as implemented in the GraFit software. Standard error bars are included.

**Figure 3:**
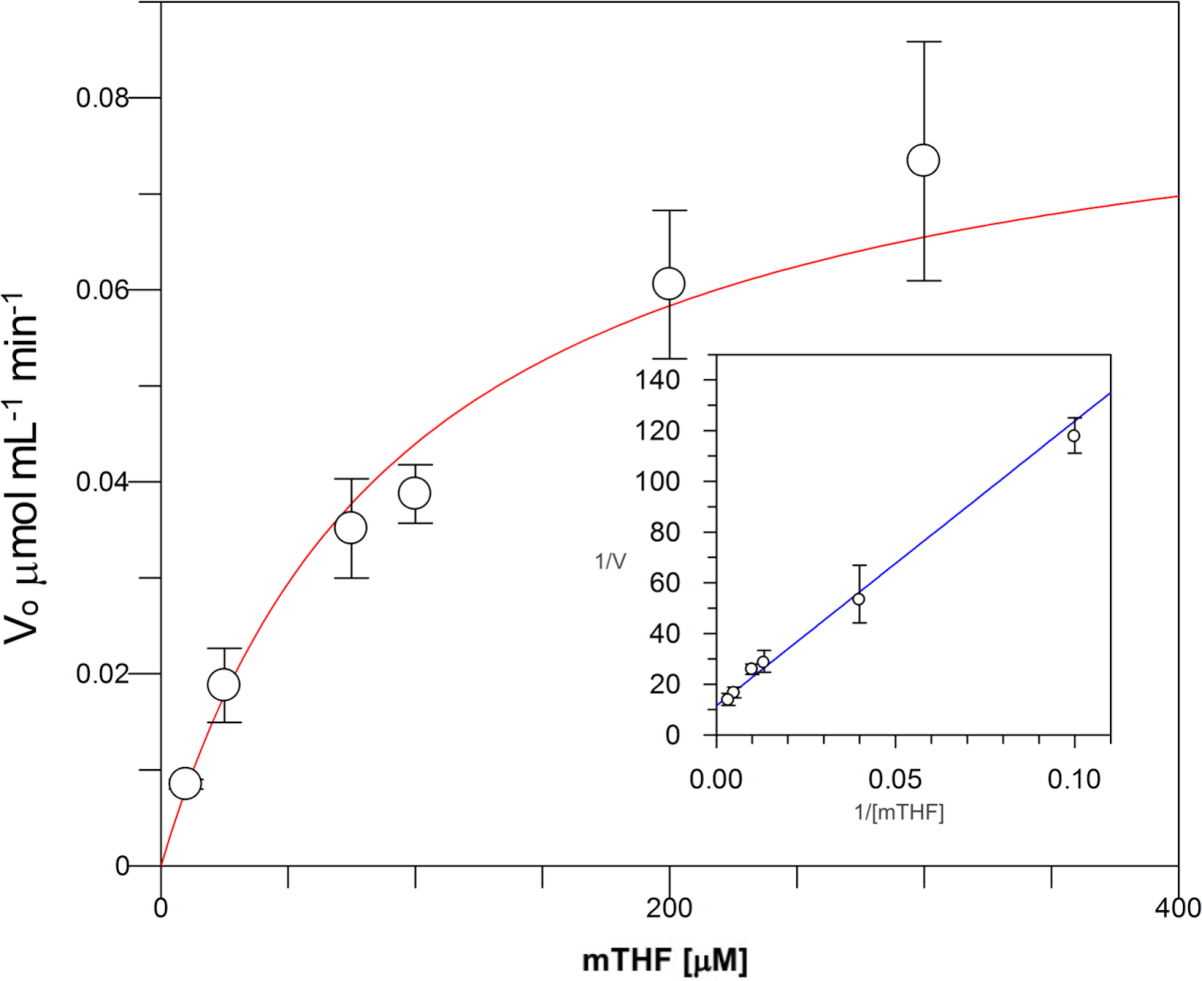
Michaelis-Menten kinetics for VpTS using mTHF as substrate. Initial velocities were measured by triplicate and kinetic parameters were obtained by non-linear fitting with robust weighting as implemented in the GraFit software. Standard error bars are included.

**Figure 4:**
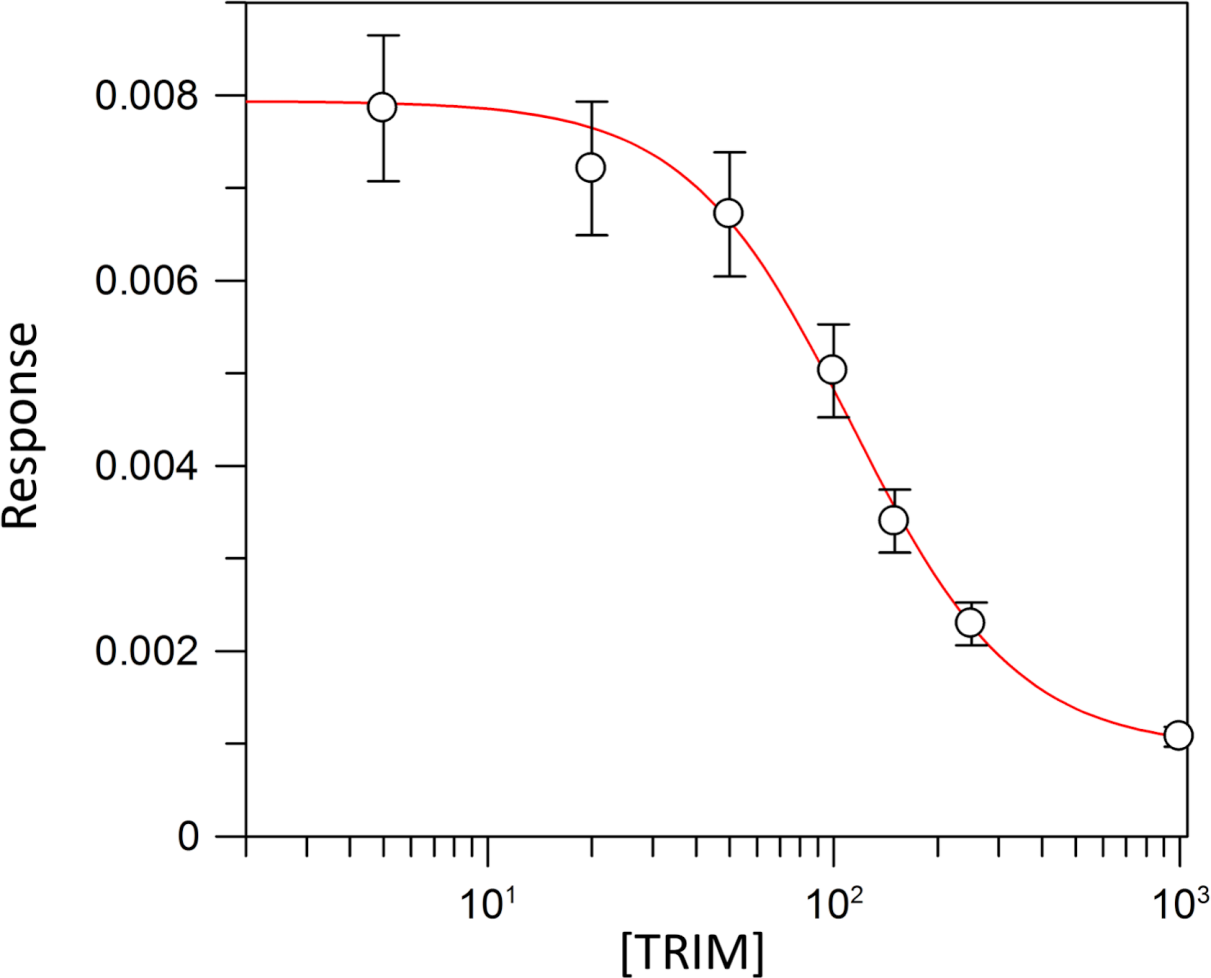
TRIM concentration for 50% inhibition (IC_50_) of the VpTS. The depicted curve describes the inhibition of TS activity, where y-axis corresponds to the initial velocity (*V*_0_) of TS, and the *x*-axis to the logarithmic TRIM concentration. Standard error bars are included.

Kinetic parameters for dUMP were 27.3 ± 4.3 µM for *K*_*m*_, 0.3 s^−1^ for *k*_*cat*_, and 0.01 µM^−1^ s^−1^for kinetic efficiency (Fig. 2). For mTHF, the values were *K*_*m*_ 96.3 ± 18 µM, *k*_*cat*_ 0.3 s^−1^, and a kinetic efficiency of 0.0031 µM^−1^ s^−1^ (Fig. 3). These values are higher than those reported for other TS in previous works (Table 1), clearly indicating that a loss of affinity occurs for both substrate and cofactor compared to prokaryotic and eukaryotic TSs (Greene et al., 1994; Spencer, Villafranca & Appleman, 1997; Fox et al., 1999; Sergeeva et al., 2003; Pozzi et al., 2012). Inhibition of VpTS was studied using TRIM, which is a broad range inhibitor of bacterial TS and dihydrofolate reductase (DHFR). Inhibition data were adjusted to a non-linear dose–response model obtaining an IC_50_ value of 106 µM, with a standard error of 11.36 µM (Fig. 4).

**Table 1:** Comparison of kinetics parameters for TS from different organisms. Standard deviation values are included where available.

Organism	*k*_*cat*_ (s^−1^)	*K*_*m*_ dUMP (µM)	*K*_*m*_ mTHF (µM)	*k*_*cat*_∕*K*_*m*_ dUMP (µM^−1^ s^−1^)	*k*_*cat*_∕*K*_*m*_ mTHF (µM^−1^ s^−1^)	
*V. parahaemolyticus*	0.3	27.3 ± 4.3	96.3 ± 18	0.01	0.003	This work
*L. vannamei*	4.1	2.1	13.5	1.95	0.030	Arvizu-Flores et al. (2009)
*WSSV*	2.8	1.2	13.4	2.33	0.209	Arvizu-Flores et al. (2009)
*L. casei*	5.1	2.6	20	1.96	0.255	Kawase et al. (2000)
*E. coli*	1.9	1.2	11	1.58	0.173	Spencer, Villafranca & Appleman (1997)
*L. lactis*	11	7.2	19	1.53	0.579	Greene et al. (1994)
*E. faecalis*	4.3	7.0	20	0.65	0.215	Pozzi et al. (2012)
*B. subtilis*	20	3.4	11.2	5.88	1.786	Fox et al. (1999)
Human	0.21	2.7 ± 0.33	10.1 ± 0.7			Sergeeva et al. (2003)

### Phylogenetic analysis of thymidylate synthases among the Gammaproteobacteria

**Figure 5:**
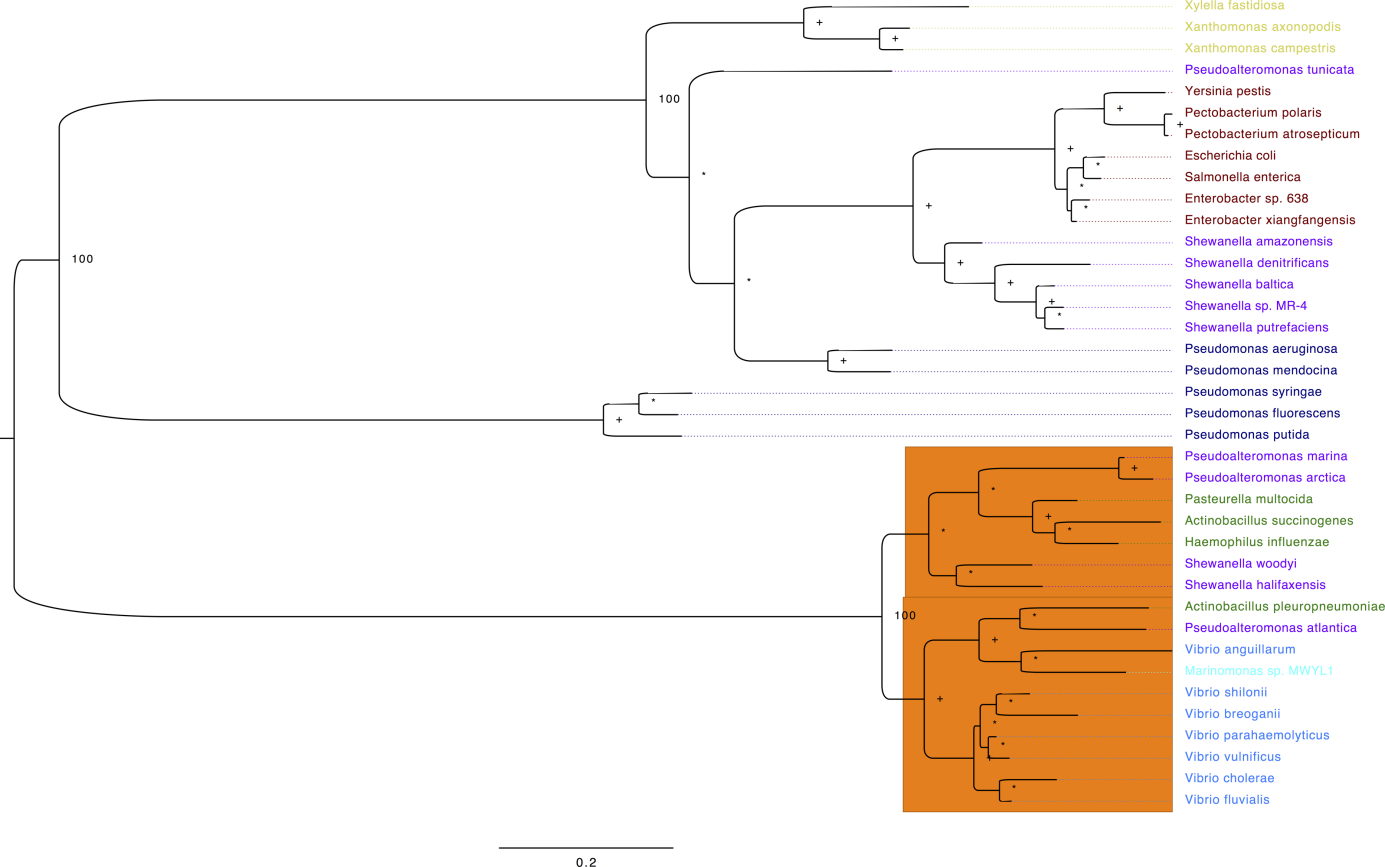
Phylogeny of TS for selected Gammaproteobacterias. Maximum likelihood phylogeny of Thymidylate synthase based on protein sequences alignment. The labels are colored corresponding to the different gammaproteobacteria orders: brown, Enterobacteriales; blue, Vibrionales; green, Pasteurellales; purple, Alteromonadales; light blue (cyan), Oceanospirales; navy blue, Pseudomonadales and Xanthomonadales in yellow. The numbers next to the nodes correspond to the bootstrap values for some of the main clades, plus signs show nodes with 70 or higher bootstrap support. Asterisks denote nodes with less than 70 bootstrap support. The orange rectangle contains all the sequences that contain a glycine in position 141 (G141) *vs*. the consensus arginine (R). The sequences used on this analysis are located in File S2.

To get a more compressive view of the molecular evolution of the TS among the gammaproteobacteria, we constructed an ML-phylogeny using 38 genomes (File S1), which represent the main orders of the Gammaproteobacteria. We have aligned selected TS amino acid sequences and highlighted in yellow the active site residues (File S2). The first thing we noticed is that two main clades were formed and the small clade (orange) corresponds to the VAAP: Vibrionales, Alteromonadales, *Pasteurellales* and Oceanospirales (Fig. 5). Interestingly, the TS of *Shewanella amazonensis*, *Shewanella denitrificans*, *Shewanella baltica*, *Shewanella sp*. MR-4, *Shewanella putrefaciens* and *Pseudoalteromonas tunicate*, do not reflect a history of the species according to the species tree of these genomes previously reported (Williams et al., 2010). These results suggest that these TS and other genes could be acquired by horizontal gene transfer in these phylogenetic clades.

Moreover, from the amino acid sequence alignment (File S2), we confirmed that all residues that form the canonical TS active site are invariant, except for the TSs in the VAAP clade (Fig. 5, orange rectangle). One of them was a conservative change of lysine to arginine at position 50 in VpTS (Table 2). This residue is important for folate binding as it makes an ionic contact between the mTHF γ-glutamate and its mutation reduces folate affinity (Arvizu-Flores et al., 2008). The other change is more dramatic, a glycine for an arginine at position 141′ in VpTS (Fig. 5, orange rectangle). This change leaves three arginines to coordinate the dUMP phosphate group and possibly reduces nucleotide affinity (Table 2). Other authors have also found that position Arg179′ (equivalent to VpTS Gly141′) was relatively permissive to mutations (Kawase et al., 2000).

**Table 2:** Function of invariant residues in TS. Selected residues with critical function for TS catalytic activity are listed for *V. parahaemolyticus*, *E. coli*, *L casei* and human sequences. Based on Finer-Moore, Santi & Stroud (2003).

*V. para.*	*E. coli*	*L. casei*	Human	Function
Cys160	Cys146	Cys198	Cys195	Catalytic residue for nuclephilic attack to dUMP
Asn191	Asn178	Asn229	Asn226	Substrate specificity towards dUMP base
His221	His207	His259	His256	Hydrogen bonding with dUMP ribose hydroxyl
Tyr223	Tyr209	Tyr261	Tyr258	*idem*
Trp82	Trp80	Trp82	Trp109	Positioning of folate
Arg22	Arg21	Arg23	Arg50	dUMP phosphate binding
Arg140′	Arg126′	Arg178′	Arg175′	*idem*
Gly141′	Arg127′	Arg179′	Arg176′	*idem*
Arg180	Arg166	Arg218	Arg215	*idem*
Arg50	Lys48	Lys50	Lys77	Ionic interaction with γ-glutamate from mTHF

### Molecular modeling of the dUMP binding site in VpTS

**Figure 6:**
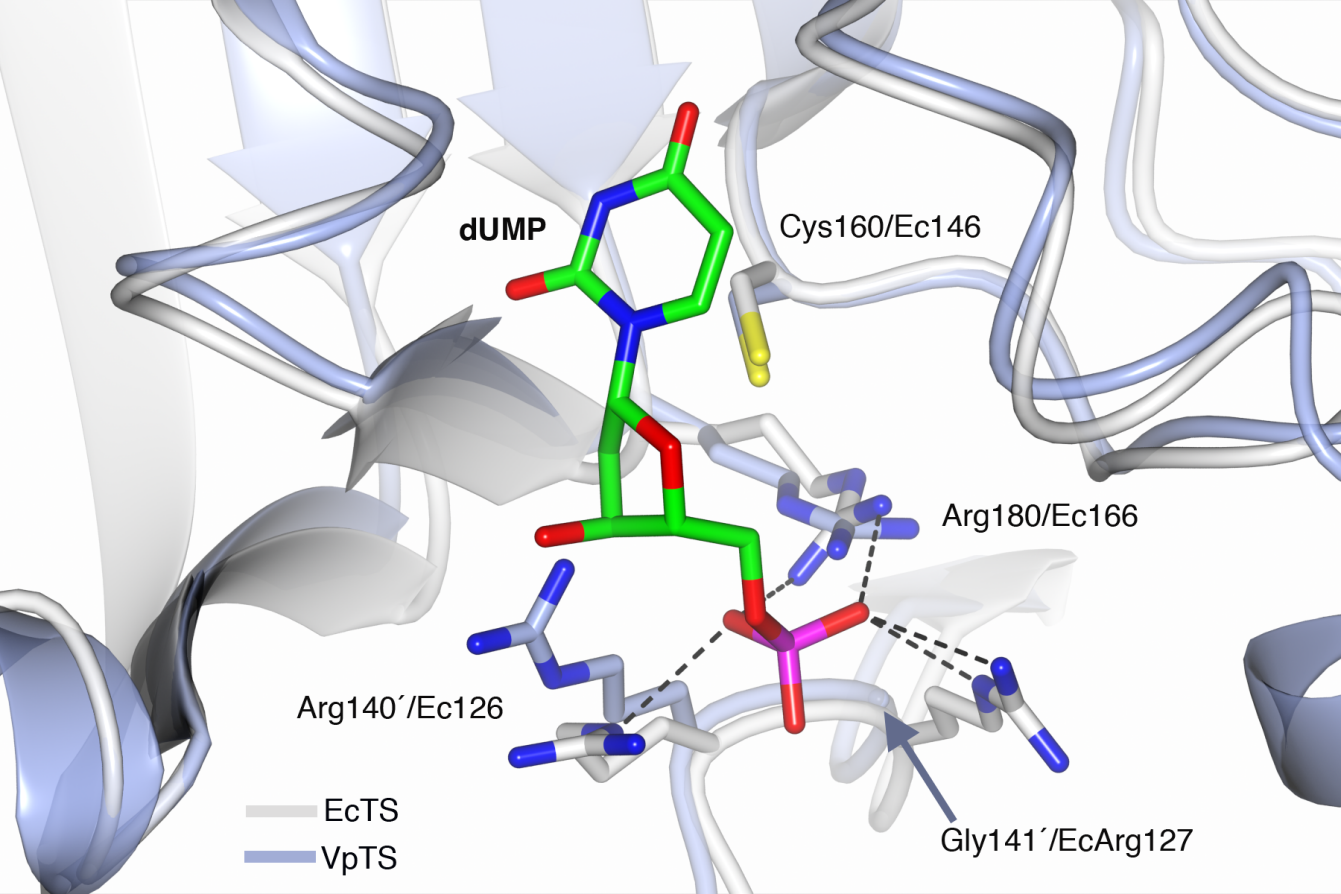
Prediction of the active site of VpTS. The theoretical model of VpTS (represented in light blue ribbons) was superimposed to the crystallographic structure of *E. coli* TS bound to dUMP (represented in light grey ribbons; PDB 1AWX). The nucleotide is represented as cylinders and colored by atom type (carbon in green, nitrogen in blue, oxygen in red, phosphorous in magenta). The catalytic cysteine and phosphate-binding arginines are included. Two invariant arginine residues VpArg180/Ec166 and VpArg140′/Ec126′ are presented. Residues from the opposite monomer have a prime (′) in their numbering. Critical sequence changes are VpGly141′/EcArg127, and an arrow is indicating the position of the alpha-carbon for VpGly141′. Hydrogen bonds are shown as dotted lines based on the crystallographic structure.

The sequence of VpTS was modeled with Phyre2 to predict the three-dimensional structure of the enzyme. TS has a well-described conformational change from an open active site when unbound or dUMP is present. A closed conformation occurs when the nucleotide and a folate analog are bound (Finer-Moore, Santi & Stroud, 2003). We modeled a dUMP-bound complex by superposing the Phyre2-VpTS model with an *E. coli* TS structure (PDB 1AXW) with a 100% confidence level and 99% alignment coverage. We confirmed that the nucleotide phosphate was coordinated by three invariant arginine residues in VpTS: Arg22, Arg 140′ and Arg180 (Fig. 6). For clarity, Arg22 was not included the figure. As mentioned above, VpTS lacks the fourth arginine residue (Ec Arg127′; Lc Arg179′; hArg176′; see Table 2) that is important to stabilize the phosphate group by two hydrogen bonds. The presence of glycine in this position (Gly141′) is recurrent in the VAAP clade (File S2).

## Discussion

The kinetic results for the VpTS are consistent with loss of one arginine residue involved in dUMP binding. Loss of affinity for the VpTS nucleotide substrate led to a reduction in catalytic efficiency compared to other TSs (Table 1). Proteins that participate directly and indirectly in the generation of nucleotides for DNA replication are highly conserved in sequence as well as in their structure (Cui et al., 2013). TS is an homodimer where each active site is comprised of residues from both monomers, although without any cooperativity (Sapienza, Falk & Lee, 2015). These differences are usually exploited in drug development for proliferative disease treatment where the pathogen and the host share the same enzyme (Carreras & Santi, 1995; De Clercq, 2002). Sequence analysis showed that VpTS has important amino acid changes concerning the consensus TSs known to this date (Table 2 and File S2).

A key change is the loss of one of the four arginine residues that coordinate the dUMP phosphate group by the presence of Gly141′ where most of TSs have an arginine residue. Site-directed mutagenesis studies in *L. casei* TS have demonstrated that changes in this position are allowed (Kawase et al., 2000), meaning that the enzyme still retains enzymatic activity.

To our knowledge, this is the first report of an organism where naturally one of the four nucleotide-binding arginines was substituted by another residue. Likewise, a change of Arg179′ for Ala, Thr, Lys or Glu, led to higher values of *K*_*m*_ in all directed-site mutants (Santi et al., 1990). In *E. coli*, Arg166 (Arg180 in VpTS) is fundamental for the thiolate formation beside nucleotide binding (Sotelo-Mundo et al., 2006). There are two other arginines that also coordinate the nucleotide phosphate, the Arg126′ and Arg 127′ in EcTS, which correspond to Arg140′ and Gly141′ in *V. parahaemolyticus,* respectively. Arg126′ has been studied structurally since a conservative mutation impairs catalysis and the structure (Strop et al., 1997).

We concluded that the increase in *K*_*m*_ for dUMP in VpTS may reflecting a loss in affinity for the nucleotide due to less favorable interactions with the dUMP phosphate. TS has an ordered kinetic mechanism, where dUMP is the first substrate bound (Spencer, Villafranca & Appleman, 1997), therefore the *K*_*m*_ for mTHF is also affected and *k*_*cat*_∕*K*_*m*_ too.

An additional change in the active site vicinity occurs at VpTS Met161, where other species have a His o Lys that makes hydrogen bonds with the uridine ring. Loss of an additional hydrogen bond by the loss of the imidazole group at position 161 could be an additional factor in a reduced nucleotide affinity. In previous works, it was shown that the changes in EcHis147, affect significantly the TS catalytic efficiency (Dev et al., 1989; LaPat-Polasko, Maley & Maley, 1990).

The conservative change at position 50 may have a steric effect over folate binding. In the T4 phage TS, the Lys48Arg mutation increased its K_*m*_ value for mTHF by two orders of magnitude respect to the wild-type enzyme. This change in size and not in charge may contribute to the increase in mTHF K_*m*_ for mTHF compared to other species (Table 1).

### TRIM inhibition of VpTS

Some treatments against proliferative, microbial, inflammatory and parasitic diseases have been focused on folate metabolism inhibition (Gonen & Assaraf, 2012). TRIM is an antifolate with structural differences respect to methotrexate, which increase their specificity for the bacterial DHFR (Navarrete et al., 2013). In this work, the inhibitory capacity by TRIM for VpTS was evaluated. The IC_50_ value was 106 µM, whereas values for bacterial DHRF are usually very low, such as 0.001 µM for *Haemophilus influenzae* and 0.01 µM for *E. coli* (Wax, 2008). Although TRIM is not a potent inhibitor of TS, due to its commercial availability, it is possible to improve its affinity to TS by rational drug design that would allow inhibition values comparable to the specific compounds for TS.

## Conclusion

In conclusion, we experimentally evaluated the biochemical properties of VpTS found in the VAAP clade that had changes in key residues for substrate binding. These changes do not imply the loss of function from site-directed mutagenesis studies done in other TSs. Nonetheless, considering that the active site of TS is invariant from phages to human, a further detail into the alternative pathway for dTMP biosynthesis such as nucleotide salvage by thymidine kinase is worth investigating. Also, the evolutionary history of the VAAP clade should deserve attention, as part of the microbiota of marine organisms and as some important pathogens such as Vibrio sp.

##  Supplemental Information

10.7717/peerj.5023/supp-1 File S1 List of genome GenBank accession number used to obtain the thymidylate synthase gene sequences

10.7717/peerj.5023/supp-2 File S2 Sequence alignment of TS amino acid sequences Members of the VAAP clade (*Vibrionales, Alteromonadales, Aeromonadales,* and *Pasteurellales*) are colored in orange. *E. coli* TS sequence was included for reference and is colored in blue. Invariant residues are highlighted in yellow across species, while positions that change in VAAP with respect to other organisms are highlighted in green. The sequence labeling on top of the alignment is based on *V. parahaemolyticus* TS. Please refer to Table 2 for an explanation of the function of these important residues.

## Additional Information and Declarations

### Data Availability

The following information was supplied regarding data availability: The sequences are available in GenBank: WP_100088861.1, JPLV00000000.1.
