# Supplementary material for: A novel thymidylate synthase from the Vibrionales, Alteromonadales, Aeromonadales, and Pasteurellales (VAAP) clade with altered nucleotide and folate binding sites
Source: PeerJ. 2018 Jun 15;6:e5023. doi: 10.7717/peerj.5023 (PMC6005164; doi:10.7717/peerj.5023)
Supplement: File S2 — Members of the VAAP clade (Vibrionales, Alteromonadales, Aeromonadales, and Pasteurellales) are colored in orange. E. coli TS sequence was included for reference and is colored in blue. Invariant residues are highlighted in yellow across species, while positions that change in VAAP with respect to other organisms are highlighted in green. The sequence labeling on top of the alignment is based on V. parahaemolyticus TS. Please refer to Table 2 for an explanation of the function of these important residues. [file peerj-06-5023-s002.pdf]

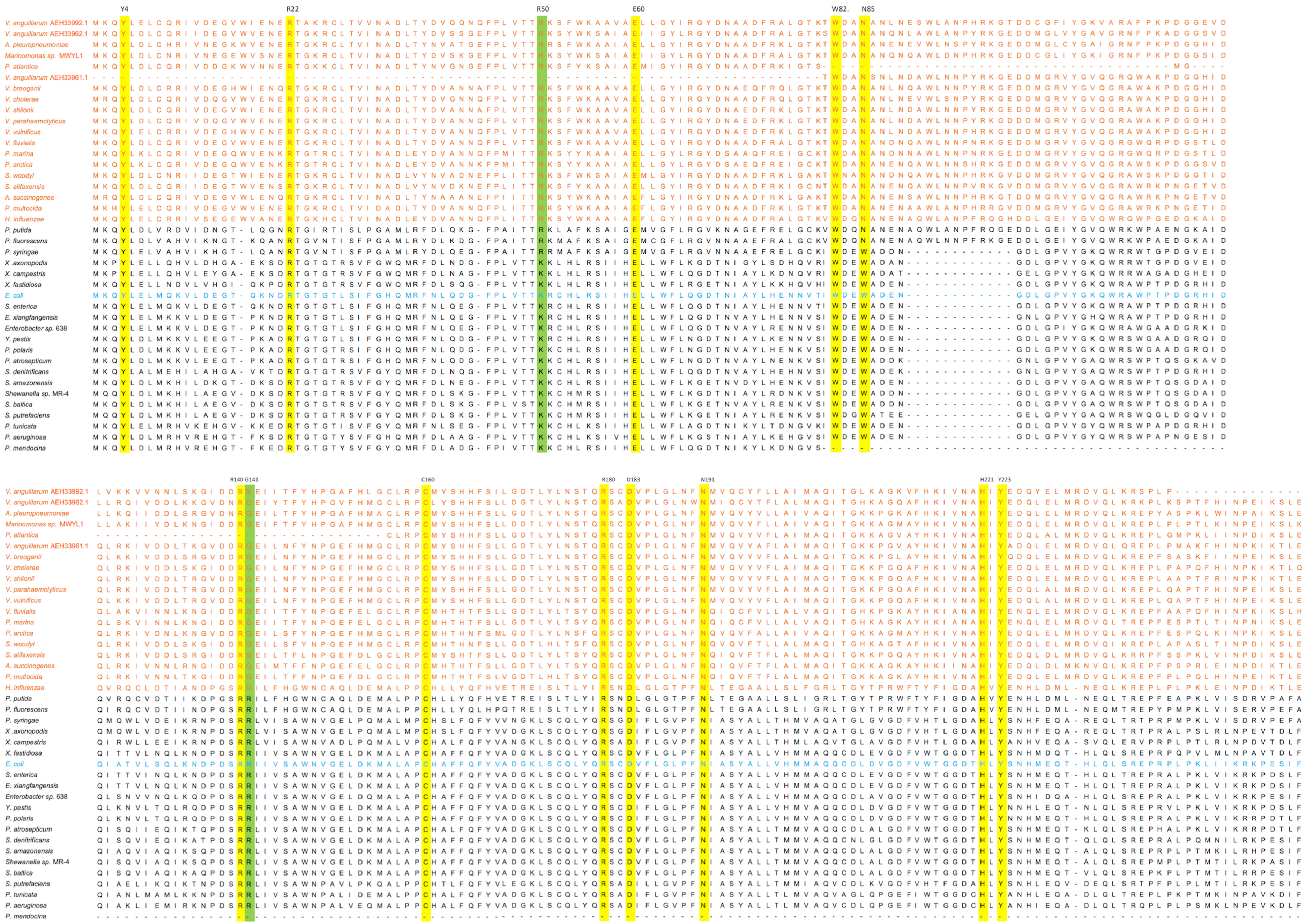
Y4
R22
R50
E60
W82.
N85
V. anguillarum AEH33992.1
V. anguillarum AEH33962.1
A. pleuropneumoniae
Marinomonas sp. MWYL1
P. atlantica
V. anguillarum AEH33961.1
V. breoganii
V. cholerae
V. shilonii
V. parahaemolyticus
V. vulnificus
V. fluvialis
P. marina
P. arctica
S. woodyi
S. alifaxensis
A. succinogenes
P. multocida
H. influenzae
P. putida
P. fluorescens
P. syringae
X. axonopodis
X. campestris
X. fastidiosa
E. coli
S. enterica
E. xiangfangensis
Enterobacter sp. 638
Y. pestis
P. polaris
P. atrosepticum
S. denitrificans
S. amazonensis
Shewanella sp. MR-4
S. baltica
S. putrefaciens
P. tunicata
P. aeruginosa
P. mendocina
R140 G141
C160
R180
D183
N191
H221
Y223
